# Radiation-Induced c-Jun Activation Depends on MEK1-ERK1/2 Signaling Pathway in Microglial Cells

**DOI:** 10.1371/journal.pone.0036739

**Published:** 2012-05-14

**Authors:** Zhiyong Deng, Guangchao Sui, Paulo Mottin Rosa, Weiling Zhao

**Affiliations:** 1 Department of Radiation Oncology and Brain Tumor Center of Excellence, Wake Forest University School of Medicine, Winston-Salem, North Carolina, United States of America; 2 Department of Cancer Biology, Wake Forest University School of Medicine, Winston-Salem, North Carolina, United States of America; University of Chicago, United States of America

## Abstract

Radiation-induced normal brain injury is associated with acute and/or chronic inflammatory responses, and has been a major concern in radiotherapy. Recent studies suggest that microglial activation is a potential contributor to chronic inflammatory responses following irradiation; however, the molecular mechanism underlying the response of microglia to radiation is poorly understood. c-Jun, a component of AP-1 transcription factors, potentially regulates neural cell death and neuroinflammation. We observed a rapid increase in phosphorylation of N-terminal c-Jun (on serine 63 and 73) and MAPK kinases ERK1/2, but not JNKs, in irradiated murine microglial BV2 cells. Radiation-induced c-Jun phosphorylation is dependent on the canonical MEK-ERK signaling pathway and required for both ERK1 and ERK2 function. ERK1/2 directly interact with c-Jun in vitro and in cells; meanwhile, the JNK binding domain on c-Jun is not required for its interaction with ERK kinases. Radiation-induced reactive oxygen species (ROS) potentially contribute to c-Jun phosphorylation through activating the ERK pathway. Radiation stimulates c-Jun transcriptional activity and upregulates c-Jun-regulated proinflammatory genes, such as tumor necrosis factor-α, interleukin-1β, and cyclooxygenase-2. Pharmacologic blockade of the ERK signaling pathway interferes with c-Jun activity and inhibits radiation-stimulated expression of c-Jun target genes. Overall, our study reveals that the MEK-ERK1/2 signaling pathway, but not the JNK pathway, contributes to the c-Jun-dependent microglial inflammatory response following irradiation.

## Introduction

Radiation therapy is an important modality of treatment for brain tumors and cancers that have metastasized to the brain. However, the side effects of radiation therapy, such as acute and chronic brain injury, are a major concern and limit its clinical application [1]. A growing body of evidence supports the hypothesis that radiation-induced brain injury is driven in part by an acute and/or chronic inflammatory response [2], [3]. As the resident innate immune cells in the brain, microglia are a predominant source of proinflammatory factors [4], [5]. Radiation stimulates the transformation of resting microglial cells to a reactive state, consequently leading to the up-regulation of proinflammatory cytokines such as tumor necrosis factor-α (TNF-α), interleukins, and cyclooxygenase-2 (COX-2) [3], [6], [7], [8], [9]. Excessive amounts of proinflammatory cytokines secreted by activated microglia can cause neurotoxicity in neurodegenerative diseases [5] and also have been implicated in radiation-induced brain injury. The release of cytokines is deleterious to neurons, since it can alter the microenvironment of neural stem and progenitor cells and may further block neurogenesis [3]. Sustained microglial activation observed in irradiated rodent brains was associated with a concomitant decline in neurogenesis in the hippocampus and spatial memory retention deficits [10], [11]. Blocking inflammation with indomethacin, a common nonsteroidal anti-inflammatory drug, inhibited radiation-induced microglial activation and mitigated impaired neurogenesis by radiation [3]. To date, the molecular mechanisms underlying radiation-stimulated microglial activation remain largely unclear.

Activation of transcription factors, such as AP-1 proteins, is one of the cellular responses to ionizing radiation [12]. c-Jun, a component of the AP-1 transcription factors, regulates expression of many genes, including inflammatory and cytokine genes, which contain consensus AP-1 binding sites in their promoter regions [13], [14], [15], [16]. Therefore, c-Jun has been considered to be an important regulator for neural cell death and neuroinflammation [17]. c-Jun generally forms homodimers or heterodimers with other AP-1 proteins and binds to specific DNA elements in promoters to regulate target genes. c-Jun protein is composed of several structural and functional domains. Its N-terminus (aa1-190) contains a c-Jun N-terminal kinase (JNK) binding domain (JBD) and a transactivation domain. The C-terminal region (aa257-308) contains a leucine zipper (bZip) type DNA binding domain. The middle part is a hinge region (aa191-256) [17]. The transcriptional activity of c-Jun is regulated by different post-translational modifications [17], [18]. Among these, phosphorylation plays a critical role in regulating c-Jun function. Phosphorylation can occur at multiple sites on the c-Jun protein [17], [19]. Phosphorylation on Ser63 and Ser73 of N-terminal c-Jun is normally associated with its enhanced transcriptional activity [17], [20]. Conversely, C-terminal phosphorylation of c-Jun decreases its ability to bind to target gene promoters [17].

c-Jun can be phosphorylated by many different types of kinases, especially mitogen-activated protein kinases (MAPKs) [21]. MAPKs belong to a Ser/Thr protein kinase family that plays a pivotal role in regulating activation of transcription factors. The MAPK cascades comprise the three-kinase module (MAPKKK, MAPKK and MAPK), and are evolutionarily conserved and ubiquitously expressed in eukaryotes [22]. Sequential activation from MAPKKK to MAPK transmits multiple cellular stimuli into nuclei to modulate gene expression and eventually initiate cellular responses. In addition to essential functions in many physiological processes including cell proliferation, senescence, differentiation, and apoptosis, MAPKs mediate the response to extracellular stimuli such as ionizing radiation and toxic stresses [21].

JNKs, ERK1/2 (p44 and p42), and p38 MAPKs represent the well characterized MAPK subfamilies. Various AP-1 proteins are regulatory targets of these MAPKs. JNKs have been recognized as the predominant c-Jun kinases targeting Ser63 and Ser73 in the c-Jun N-terminus; whereas ERK1/2 and p38 are critical for c-fos phosphorylation [22]. Each member of the MAPK superfamily kinases is involved in distinct signal cascades, respectively; however, they are also functionally related in certain circumstances. A recent study has demonstrated that ERK1/2 functionally substituted for JNK1/JNK2 to mediate phosphorylation of Ser63/73 on c-Jun protein in vitro and in mouse fibroblasts [19], indicating a crosstalk between JNK and the ERK1/2 signaling pathways. However, the functional relationship between the ERK1/2 cascade and c-Jun function still remains largely undetermined.

In this study, we used BV2 murine microglial cells as a model to explore the molecular signaling pathway required for c-Jun activation in response to ionizing radiation. We discovered that the canonical MAPK MEK-ERK1/2 signaling cascade, but not JNK pathway, directly mediated c-Jun N-terminal phosphorylation. Blockade of MEK-ERK1/2 signaling pathway compromised c-Jun activity, leading to a decreased expression of several proinflammatory genes regulated by c-Jun.

## Results

### Ionizing radiation leads to a rapid phosphorylation of c-Jun and ERK1/2, but not JNKs

**Figure 1:**
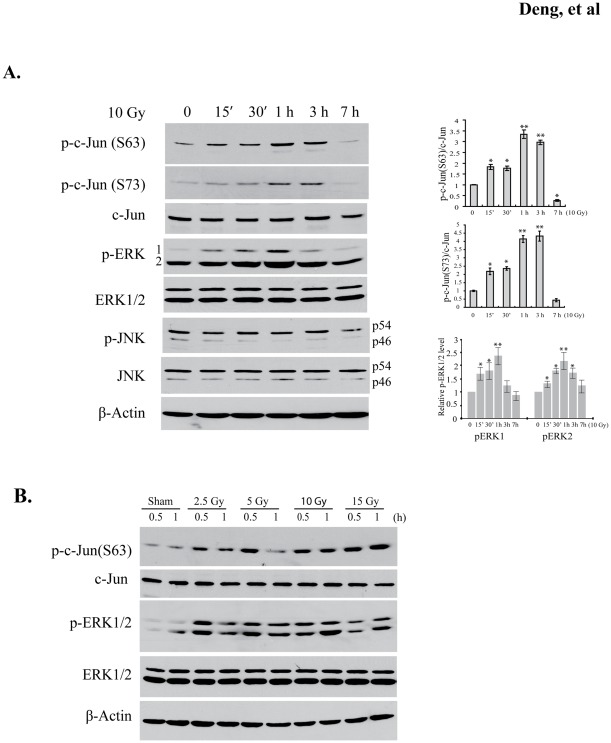
Radiation-induced phosphorylation of c-Jun and ERK1/2 in BV2 cells. (A) BV2 cells were irradiated with a single dose of 10 Gy, and sampled at the time points indicated. Cell lysates were sequentially immunoblotted with indicated antibodies in Western blot assays to detect phosphorylated and total proteins of c-Jun, ERK1/2, and JNK. β-actin was used as a loading control. Increased phosphorylation was found for c-Jun and ERK1/2 post irradiation, but not for JNKs. There was no obvious change for total c-Jun. The right panel shows the fold change for phosphorylation of c-Jun (Ser63 and Ser73) and ERK1/2 following irradiation. Data represent mean ± SD and the significant differences compared to control were indicated as *p<0.05 or **p<0.01. (B) BV2 cells were irradiated with different doses as shown and phosphorylation of c-Jun and ERK1/2 was detected at 0.5 and 1 h post-irradiation. Regardless of dose levels, radiation markedly stimulated phosphorylation of c-Jun and ERK1/2.

BV2 cells were irradiated with a single dose of 10 Gy γ-ray and cell lysates were collected for Western blot analyses. Radiation resulted in a prominent increase in c-Jun phosphorylation on Ser63 and Ser73 (Figure 1A), the critical phosphorylation sites correlating with enhanced transcriptional activity of c-Jun [23]. The induction of phosphorylation by radiation was rapid and sustained for several hours. Meanwhile, total c-Jun protein remained unchanged in the irradiated cells, indicating that the accumulation of phosphorylated c-Jun was not due to transcriptional upregulation. JNKs have been known to phosphorylate c-Jun on Ser63 and Ser73 residues. Unexpectedly, we did not observe concurrently activated JNKs following irradiation, indicated by their unchanged phosphorylation status on Thr183 and Tyr185 sites (Figure 1A). In contrast, radiation stimulated rapid phosphorylation of ERK1 and ERK2. Similar results were found in BV2 cells treated with different irradiation doses from 2.5 to 15 Gy. As shown in Figure 1B, phosphorylation was increased under each of the conditions for both c-Jun and ERK1/2. Nevertheless, induction patterns of the phosphorylation were somewhat different between low-dose and high-dose treated cells. For example, peak phosphorylation for c-Jun and ERK1/2 appeared at 0.5 h for lower doses of irradiation (2.5 and 5 Gy), but at 1 h for the highest dose (15 Gy). Moreover, there was an apparent correlation between post-irradiation phosphorylation levels of c-Jun and ERK1/2, suggesting that ERK1/2 mediate radiation-stimulated c-Jun phosphorylation.

### ERK1 and ERK2 are required for radiation-induced c-Jun phosphorylation in BV2 cells

**Figure 2:**
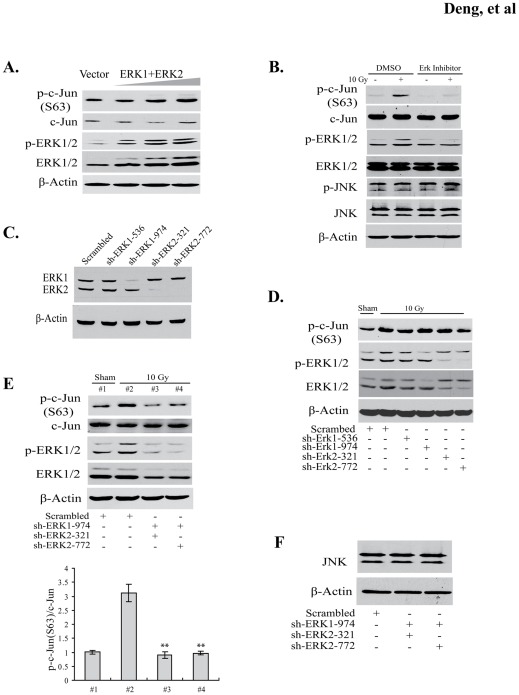
ERK1 and ERK2 are indispensable for radiation-induced c-Jun phosphorylation in BV2 cells. (A) BV2 cells were transiently cotransfected with ERK1 and ERK2 expression vectors in doses of 75, 150 and 300 ng for each plasmid. The total DNA amounts in transfection were compensated with an empty vector. The transfected ERK1 and ERK2 led to a dose-dependent increase of phosphorylated ERK1/2 and c-Jun. (B) BV2 cells were treated with 10 µM ERK kinase inhibitor (Calbiochem Cat #328006) and DMSO as a control. Inhibitor-treated or untreated cells were collected at 1 h after 10 Gy irradiation. Western blots showed that ERK kinase inhibitor abolished radiation-stimulated phosphorylation of ERK1/2 and c-Jun. (C) To detect knockdown effects for ERK1 and ERK2 by lentiviral shRNA constructs in BV2 cells, two lentivirual shRNAs were prepared for each ERK kinase, respectively, and used for infecting BV2 cells. The cells were collected 48 h after lentiviral infection and subjected for Western blot analysis using the antibody against ERK1 (K32, sc-94), which also can recognize ERK2. Efficient depletion was found in the cell lysates infected with sh-ERK1-974, sh-ERK2-321, and sh-ERK2-772, but not sh-ERK1-536. (D) BV2 cells were individually infected with scrambled control, ERK1, and ERK2 shRNA lentiviruses, and subsequently irradiated with 10 Gy 48 h post infection and sampled at 1 h following radiation. There was no effect on radiation-induced c-Jun phosphorylation by individual depletion of ERK kinases. (E) BV2 cells were infected with combined lentiviruses (sh-ERK1-974/sh-ERK2-321 and sh-ERK1-974/sh-ERK2-772) and irradiated (10 Gy). Both shRNA combinations reduced radiation-stimulated phosphorylation of ERK1 and ERK2 to low levels, and decreased phosphorylated c-Jun after radiation. Bar graph depicts quantification (means ± SD) of relative c-Jun phosphorylation level, **p<0.01(#3 vs. #2; #4 vs. #2). (F) JNK levels in ERK1/2 knockdown BV2 cells.

To determine whether ERK1 and ERK2 regulate c-Jun phosphorylation in BV2 cells, *ERK1* and *ERK2* expression vectors were cotransfected into BV2 cells in increasing amounts to overexpress these two proteins. Forty-eight hours after transfection, c-Jun phosphorylation on Ser63 showed a dose-dependent increase, indicating the capability of ERK1/2 to stimulate c-Jun phosphorylation (Figure 2A). To further substantiate the role of ERK1/2 in radiation-induced c-Jun phosphorylation, an inhibitory assay was performed. The ERK inhibitor was found to attenuate phosphorylation of ERK1/2 but not JNK kinases and accordingly markedly reduced radiation-mediated phosphorylation of Ser63 on c-Jun (Figure 2B).

We did not observe any effects of ERK inhibitor on the activity of JNKs (Figure 2B). Nevertheless, to exclude other potential non-specific effects of this inhibitor and determine which ERK isoform is more important for c-Jun phosphorylation, we conducted shRNA-mediated gene knockdown experiments for ERK1 and ERK2. Two lentiviral shRNA constructs with different target sequences were created for each of the kinases. Except for sh-ERK1-536, the other three constructs (sh-ERK1-974, sh-ERK2-321, and sh-ERK2-772) led to a specific knockdown for their corresponding targets, respectively (Figure 2C). Consistently, these effective shRNA constructs almost eliminated radiation-induced phosphorylation of ERK1 and ERK2 in BV2 cells (Figure 2D). However, individual infection with these constructs did not decrease radiation-induced c-Jun phosphorylation (Figure 2D).

To determine whether ERK1 and ERK2 have functional redundancy in mediating c-Jun phosphorylation, we simultaneously silenced these two kinases using the shRNA combinations of sh-ERK1-974/sh-ERK2-321 and sh-ERK1-974/sh-ERK2-772. Both shRNA combinations markedly decreased protein levels of ERK1 and ERK2 as well as their phosphorylation in the irradiated cells (Figure 2E). Accordingly, depletion of both ERK1 and ERK2 suppressed radiation-stimulated phosphorylation of c-Jun (Figure 2E). The reduced c-Jun phosphorylation was not due to JNK protein function, since the ERK kinase shRNAs did not affect JNK expression (Figure 2F). It is noteworthy that ERK1/2 silencing only reduced c-Jun phosphorylation to the levels comparable to that of non-irradiated control cells, suggesting that the basal level of c-Jun phosphorylation is maintained by other kinases, such as JNKs.

### ERK1 and ERK2 interact with c-Jun

**Figure 3:**
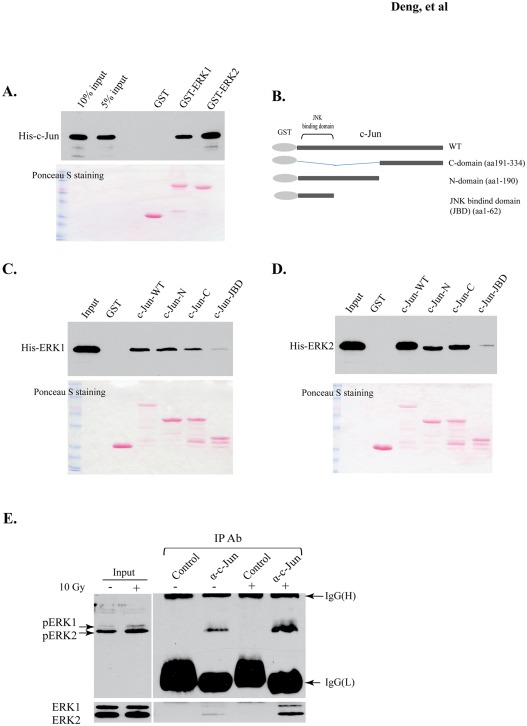
ERK1 and ERK2 interact with c-Jun in vitro and in cells. (A) Direct protein interaction between c-Jun and ERK1/2. In GST pull-down binding assays, purified 6xHis-c-Jun protein (1.0 µg) was individually incubated with GST-ERK1 and GST-ERK2 containing beads (3 µg each). After extensive washing, an antibody (sc-45) against c-Jun was used to detect the 6xHis-c-Jun protein brought down from the glutathione agarose beads. Lower panel: Ponceau S-stained membrane transferred with the GST fusion proteins. (B–D) *In vitro* protein binding studies to determine the binding domains of ERK1/2 on c-Jun. (B). Schematic diagram of GST-fusion proteins created for wild-type c-Jun and truncated c-Jun including aa191-334 (DNA binding domain), aa1-190 (JBD and transactivation domains), and aa1-62 (JBD domain). (C) and (D) Binding assays for GST-c-Jun mutants with 6xHis-ERK1 and 6xHis-ERK2. (E) Interaction of endogenous c-Jun and phosphorylated ERK kinases in BV2 cells. BV2 cells were serum starved for 24 h and then irradiated or sham-irradiated with a single dose of 10 Gy. Cell lysates were collected 1 h after irradiation. 0.75 mg cell lysates were used in co-immunoprecipitation with the c-Jun antibody (sc-45). The phosphorylated and total ERK1/2 were detected with the antibody sc-81492 and sc-94, respectively.

Upon phosphorylation, the cytosolic ERK1/2 proteins translocate into the nucleus to modulate many nuclear targets, including transcription factors [22]. To support our hypothesis that ERK1/2 directly phosphorylate c-Jun in irradiated cells, we looked for evidence of physical interaction between c-Jun and ERK1/2. In GST pull-down assays, the bacterially expressed 6xHis tagged c-Jun was purified and individually incubated with the beads containing GST-ERK1, GST-ERK2, and GST (as a control). As shown in Figure 3A, both GST-ERK1 and GST-ERK2 could specifically pull down His-tagged c-Jun protein, with ERK2 showing a higher binding affinity than ERK1.

The JBD domain of c-Jun is required for interacting with JNKs [17]. Although ERK1/2 and JNKs have been classified in the same kinase family, it is still unclear whether they bind to the same region on c-Jun protein. Thus, we created several constructs expressing GST-fused truncation mutants that contain different c-Jun domains (Figure 3B). In GST pull-down assays, purified protein of 6xHis-ERK1 or 6xHis-ERK2 was incubated with GST tagged wild-type c-Jun and its mutants (Figure 3C, 3D), respectively. Both ERK1 and ERK2 showed a similar weak interaction with the mutant (aa1-62) containing the JBD region; however, they efficiently bound to either N- (aa1-190) or C- (aa191-334) terminal c-Jun, revealing a binding pattern different from that of JNKs. Results of protein binding assays suggest that ERK1/2 may potentially mediate JNK-independent c-Jun activation.

We overexpressed Flag-c-Jun and HA-ERK1 or HA-ERK2 in BV2 cells and observed that both HA-ERK1 and HA-ERK2 were associated with Flag-c-Jun in a co-immunoprecipitation assay (Figure S1). To further examine the association of endogenous c-Jun and ERK1/2, we used a c-Jun specific antibody to immunoprecipitate the BV2 cell lysate and then examined the phosphorylated and total ERK1/2 in the precipitated proteins by Western blots. As shown in Figure 3E, c-Jun antibody brought down phosphorylated and total ERK proteins in both non-irradiated and irradiated BV2 cells. The precipitated ERK1/2 were increased in irradiated BV2 cell lysates compared with control samples, most likely due to the increased nuclear ERK1/2 (phosphorylated ERK1/2) stimulated by radiation. The higher level of precipitated phorsphorylated ERK2 compared to ERK1 is likely due to the different amounts of their phosphorylated forms in BV2 cells as well as the relatively higher binding affinity of c-Jun to ERK2 than ERK1.

### MEK-ERK1/2 signaling pathway is responsible for radiation-induced c-Jun phosphorylation

**Figure 4:**
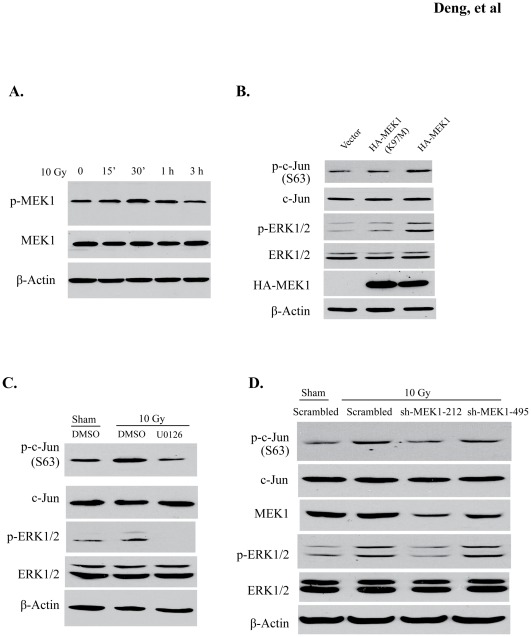
MEK-ERK1/2 signaling pathway is required for radiation-induced c-Jun phosphorylation. (A) Cell lysates collected from 10 Gy-irradiated BV2 cells were immunoblotted with anti-phosphorylated MEK1/2 (sc-7995R) antibody. (B) BV2 cells were transfected with HA-MEK1, HA-MEK1^K97M^ (kinase-dead mutant), and vector control. Protein levels of p-c-Jun and p-ERK1/2 (upper and lower panels) are shown. (C) BV2 cells that were treated or untreated with 10 μM U0126 were irradiated with 10 Gy. p-c-Jun and p-ERK1/2 levels were analyzed by Western blot. (D) Two shRNAs (sh-MEK1-212 and sh-MEK1-495) were lentivirally delivered into BV2 cells to knockdown MEK1 protein. Knockdown efficiency and protein levels of p-c-Jun and p-ERK1/2 were detected with the indicated antibodies. The quantification results for (C) and (D) are shown in Figure S2.

As the last downstream kinases in the Ras-Raf-MEK-ERK1/2 MAP kinase cascade, ERK1 and ERK2 are directly activated by MEK [21]. To confirm that radiation-induced c-Jun phosphorylation is dependent on the activation of this MAP kinase signaling pathway, we examined activation of MEK1 in irradiated BV2 cells. As shown in Figure 4A, radiation rapidly stimulated MEK1 phosphorylation. To validate the capability of MEK1 in modulating c-Jun phosphorylation, HA-tagged wild type (WT) MEK1 and its kinase-dead mutant MEK1^K97M^ [24] were overexpressed in BV2 cells by transient transfection. As shown in Figure 4B, MEK1 (WT) dramatically induced phosphorylation of ERK1/2 as well as c-Jun, while the vector or MEK1^K97M^ did not increase c-Jun phosphorylation. We also treated the BV2 cells with U0126, a MEK1 inhibitor, and observed markedly decreased phosphorylation of ERK1/2 and c-Jun in the irradiated cells (Figure 4C, and Figures S2A and S2B). To specifically disrupt MEK1 function, two lentiviral shRNAs constructs (sh-MEK1-212 and sh-MEK1-495) with different target sequences on MEK1 were generated. As illustrated in Figure 4D, and Figures S2C and S2D, sh-MEK1-212 efficiently depleted MEK1 protein level in BV2 cells and concurrently reduced radiation-stimulated phosphorylation of both ERK1/2 and c-Jun. By contrast, the sh-MEK1-495 construct, which marginally decreased MEK1, did not block radiation-increased phosphorylation of ERK1/2 and c-Jun. Collectively, these results indicate that MEK1 is indispensable for N-terminal c-Jun phosphorylation in response to radiation. Our data also suggest that the canonical MEK1-ERK1/2 signaling pathway contributes to radiation-induced c-Jun activation.

### JNK function on radiation-induced phosphorylation of c-Jun

**Figure 5:**
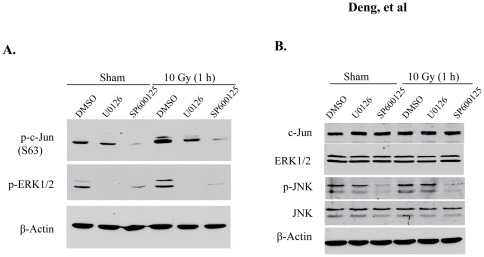
JNK function on c-Jun phosphorylation in BV2 cells. BV2 cells were individually treated with U0126 (10 μM) and the JNK inhibitor SP600125 (5 μM). Cells were irradiated (10 Gy) or sham-irradiated. Cell lysates were collected 1 h post-irradiation. (A) Levels of p-c-Jun and p-ERK1/2 in the treated cells. (B) Levels of c-Jun, ERK, p-JNKs, and JNKs in the treated cells.

The members of JNK family are primary kinases responsible for N-terminal phosphorylation of c-Jun under different physiological conditions in multiple cell types [17], [22]. A recent study showed that the JNK inhibitor SP600125 could reduce c-Jun phosphorylation in BV2 cells post-irradiation [25]. However, we did not observe JNK activation in irradiated BV2 cells, suggesting that JNKs may not be involved in modulation of c-Jun phosphorylation following irradiation [25]. To reconcile these conflicting findings, we treated BV2 cells with SP600125 and used the MEK inhibitor U0126 as a control. We indeed detected the inhibitory effect of SP600125 on c-Jun phosphorylation induced by radiation (Figure 5A); however, the JNK inhibitor SP600125 also reduced ERK1/2 phosphorylation (Figure 5A). We concluded that this unexpected effect could contribute to the reduced c-Jun phosphorylation reported previously [25]. Between these two kinase inhibitors, U0126 exhibited much better inhibition on ERK1/2 phosphorylation, but SP600125 showed a greater effect in reducing c-Jun phosphorylation (Figure 5A). These results suggest that JNKs, rather than ERK1/2, are key factors for basal c-Jun phosphorylation. Due to its dual inhibitory roles in JNK and ERK1/2, SP600125 would likely reduce both basal and radiation-induced phosphorylation of c-Jun. Indeed, we found that SP600125 was more potent than U0126 in reducing c-Jun phosphorylation following irradiation (Figure 5A). Further studies validated that JNK phosphorylation was decreased by SP600125, but not U0126, in this inhibitor assay (Figure 5B). Overall, our observations strongly suggested that radiation stimulates JNK-independent c-Jun phosphorylation via an activated ERK1/2 pathway, while JNKs are critical for c-Jun phosphorylation in other physiological conditions.

### Radiation-induced c-Jun phosphorylation is mediated by ROS

**Figure 6:**
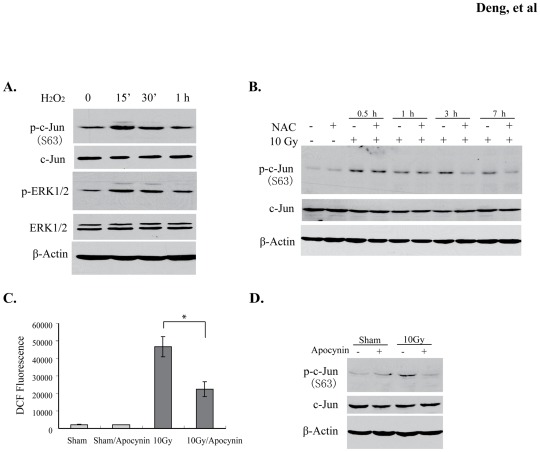
ROS is associated with c-Jun phosphorylation. (A) 100 μM exogenous hydrogen peroxide was added into culture medium of BV2 cells. p-c-Jun and p-ERK1/2 were detected at the time points indicated. (B) c-Jun phosphorylation was detected for BV2 cell lysates treated or untreated with 5 mM NAC at the indicated time points after radiation. (C) Inhibition of radiation-induced ROS in BV2 cells by 50 μM apocynin. Intracellular ROS level was determined using the 2′, 7′-Dichlorofluorescein diacetate (DCFA-DA) based method. Data represent mean ± SD, *p<0.05. (D) Apocynin was added into the BV2 cell culture medium and phosphorylation of c-Jun was analyzed in the apocynin-treated cell lysates.

Cellular oxidative stress is one of primary consequences caused by irradiation. We and others have observed increased generation of reactive oxygen species (ROS) following radiation in various cells, including microglia [25], [26], [27]. To test whether c-Jun phosphorylation is dependent on increased generation of ROS by irradiation, we treated BV2 cells with 100 µM H_2_O_2_ and observed that phosphorylated ERK1/2 and c-Jun were increased in BV2 cells following H_2_O_2_ treatment (Figure 6A). Conversely, we found that the treatment with 2.5 mM NAC, an antioxidant, attenuated the c-Jun phosphorylation in a time-dependent manner in irradiated BV2 cells (Figure 6B), revealing a delayed effect associated with a gradual ROS decrease by this oxidant scavenger. It is known that electron transport chain and NADPH oxidases are generators of ROS following irradiation [2], [26]. We therefore tested several inhibitors to study the relationship between this type of ROS generation and c-Jun activation. However we only found that apocynin, an inhibitor of NADPH oxidases, could be used in this study, because other tested inhibitors, such as TTFA, rotenone, NaN_3_, and Dpi, could nonspecifically affect c-Jun phosphorylation in non-irradiated BV2 cells (data not shown). After preincubating BV2 cells with apocynin, we observed a marked reduction of radiation-induced ROS in these cells (Figure 6C). Importantly, apocynin treatment also decreased c-Jun phosphorylation in response to irradiation (Figure 6D), suggesting the importance of ROS on radiation-stimulated c-Jun function.

### Radiation-induced phosphorylation of c-Jun modulates its transcriptional activity

**Figure 7:**
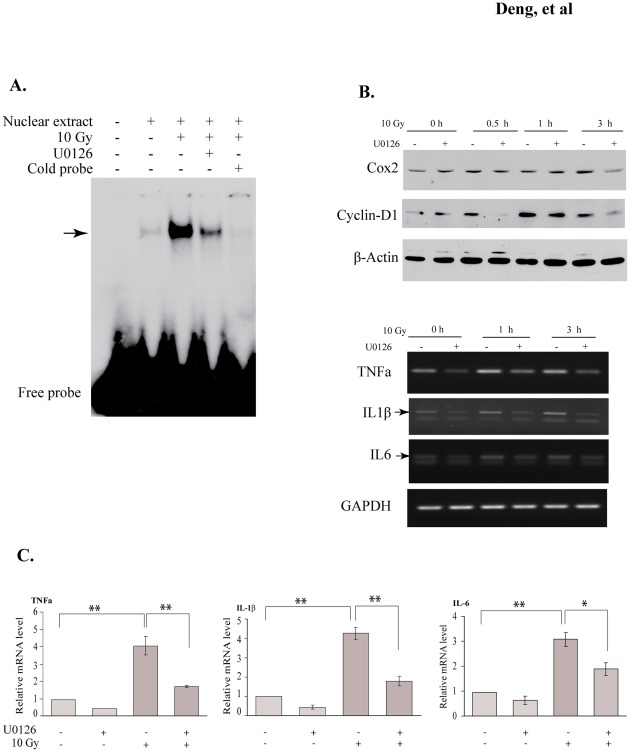
Radiation-induced phosphorylation of c-Jun modulates its transcriptional activity. (A) Radiation-increased AP-1 DNA binding ability was reduced by MEK kinase inhibitor U0126. To determine the effect of U0126 on c-Jun DNA binding ability, different nuclear extracts as indicated were incubated with the γ-^32^p-labeled AP-1 consensus DNA probe. 5% acrylamide gel (0.5× TBE) was used to resolve the products of the binding reaction. Binding specificity is shown by the reaction only with BSA and competition with 50-fold excess non-radioactive consensus AP-1 oligonucleotide (cold AP-1 oligo). (B) BV2 cells were irradiated with 10 Gy dose with or without U0126. Cells were collected at the indicated time points and subsequently used to detect the expression of c-Jun target genes. Protein levels of COX-2 and cyclin D1 were examined by Western blot and transcription levels of TNFα, IL-1β, and IL-6 were examined by RT-PCR. (C) Real-time PCR was used to quantitatively analyze the expression of TNFα, IL-1β, and IL-6. Data represent mean ± SD, *p<0.05; **p<0.01.

To validate the importance of c-Jun phosphorylation on Ser63 and Ser73 in BV2 cells, we conducted a Gal4/UAS Gaussia luciferase assay [28], [29] and found that both Ser63 and Ser73 were critical for c-Jun transcriptional activity in this cell line (Figure S3). c-Jun phosphorylation on Ser63/73 can stimulate its transcriptional activity as well as DNA-binding ability [30], [31]. To determine whether the radiation-activated ERK1/2 signaling pathway modulates c-Jun transcriptional function, c-Jun DNA binding ability was examined using a EMSA assay. We individually incubated the nuclear extracts isolated from BV2 cells with γ-^32^P labeled AP-1 probes. As shown in Figure 7A, radiation caused a substantially high level of the retarded AP-1 probe, suggesting enhanced DNA binding ability of c-Jun. Meanwhile, the inhibitor U0126 markedly diminished radiation-mediated c-Jun DNA binding ability (Figure 7A).

To further substantiate the role of ERK signaling pathway on c-Jun transcriptional activity, we studied several well-characterized c-Jun target genes, including four proinflammatory genes (COX-2, TNFα, IL-1β, and IL-6) and cyclin D1[13], [32], [33], [34], [35] in irradiated BV2 cells. We measured the expression of these downstream genes by Western blots or RT-PCR. As shown in Figure 7B, the expression of all these genes was induced by irradiation. Additionally, inhibition of the ERK signaling pathway led to decreased expression of these tested genes, consistent with the observation in c-Jun DNA binding assay. In the quantitative real-time PCR experiments, these proinflammatory genes were significantly induced by irradiation and their induction was significantly inhibited by blockade of the ERK signaling pathway (Figure 7C).

In summary, we proposed a working model (Figure S4) based on these findings. As depicted in this model, radiation-mediated oxidative stress stimulates the MEK-ERK1/2 kinase cascade and consequently activates c-Jun transcriptional function, leading to the inflammatory response in microglial cells.

## Discussion

Owing to its functional significance, c-Jun activation is subtly regulated by various post-transcriptional modifications in cells, such as phosphorylation, which have profound impacts on c-Jun function. c-Jun is phosphorylated on multiple sites in different cellular processes [36], [37], [38], [39], [40], [41], mainly on its serine and threonine residues. Phosphorylation can occur on the Ser63, Ser73, Thr91, and Thr93 sites within the transactivation domain located on the c-Jun N-terminus. In addition, residues located proximal to the C-terminal DNA-binding domain, such as Thr231, Thr239, Ser243, and Ser249, are also targeted by different kinases, respectively [19]. N-terminal phosphorylation determines whether c-Jun is transcriptionally active [17], [23]; by contrast, phosphorylation on other regions of c-Jun may impair its DNA-binding ability, leading to a compromised transcriptional activity [17], [42]. The N-terminal residues Ser63 and Ser73 on c-Jun protein are more functionally important and better characterized than other phosphorylation sites. The phosphorylation of these two serines significantly activates c-Jun transcriptional activity [17] and can be induced by various stimuli, such as lipopolysaccharide (LPS), TNFα, phorbol ester (TPA), EGF, and ultraviolet light, which are related to multiple biological processes [19], [43]. In this study, we reported that c-Jun was rapidly activated through Ser63/Ser73 phosphorylation in irradiated microglial cells. Our study implicates that radiation-stimulated c-Jun activation correlates with increased expression of proinflammatory factors and may potentially lead to microglial activation in brain.

In the mammalian kinome, MAPKs are predominantly responsible for c-Jun phosphorylation on Ser63 and Ser73. JNKs were initially identified and are generally recognized as the preferential enzymes for c-Jun N-terminal phosphorylation, especially on Ser63 and Ser73. JNK kinases bind to a small portion of the c-Jun N-terminus and consequently exert their kinase function on c-Jun [17]. In addition to JNKs, other MAPKs were also found to regulate c-Jun phosphorylation in different cellular processes. The requirement of different subtypes of MAPKs may depend on cell type and/or cellular context. In T-lymphocytes, P38 mediates a specific function for c-Jun phosphorylation in a JNK-independent manner [39]. In mouse fibroblasts isolated from JNK-deficient mice, the ERK1/ERK2 pathway substituted for JNKs to mediate TPA- and EGF-induced phosphorylation of c-Jun on Ser63 and Ser73 [19]. Moreover, ERK1 and ERK2 directly phosphorylated c-Jun on Ser63 and Ser73 in vitro [19], [44]. Despite these findings, the physiological roles of ERK1/2 on c-Jun phosphorylation have rarely been addressed.

Our study indicated that the ERK1/2, but not the JNKs, were rapidly phosphorylated in microglial cells after irradiation. We provided multiple lines of evidence using overexpression, pharmacological inhibitor, and gene knockdown approaches to prove that both ERK1 and ERK2 are indispensable for radiation-induced c-Jun phosphorylation on Ser63 and Ser73. In accordance with this novel discovery, ERK1 and ERK2 directly interact with c-Jun in vitro, and their phosphorylated forms are endogenously associated with c-Jun in cells. Interestingly, ERK2 was more efficient than ERK1 in binding to c-Jun in vitro, although they are highly homologous to each other. Meanwhile, the phosphorylated form of ERK2 is more abundant than that of ERK1 in BV2 cells, as indicated by Western blots. However, it seems that these differences do not make ERK2 more important in mediating c-Jun phosphorylation, since individual knockdown of these two kinases was insufficient in blocking radiation-induced c-Jun phosphorylation in our study, suggesting that ERK1 and ERK2 are functionally compensating each other in regulating c-Jun. Despite functional resemblance between ERK1/2 and JNKs, discrepancies exist between these two subtypes of MAPKs in their binding pattern with c-Jun. The N-terminal c-Jun contains the JBD region that is indispensable for JNK-mediated c-Jun phosphorylation. However, the c-Jun mutant (aa1-62) containing JBD region showed a weaker binding ability to ERK1 and ERK2, compared to other c-Jun truncation mutants. Our mapping study could not further define a specific ERK-binding domain on c-Jun, since it appears that multiple regions of c-Jun are likely involved in its interaction with ERKs.

ERK 1 and ERK2 are autophosphorylable on both threonine and tyrosine residues and the autophosphorylation correlates with their activation [45]. However, our results suggest that the autophosphorylation may not play an important role in their radiation-stimulated activation, due to the dependence of MEK1 function. Our data demonstrated that MEK1 can be activated in response to irradiation. More importantly, MEK1 knockdown led to the expected consequences, such as reduced phosphorylation of both ERKs and c-Jun in irradiated BV2 cells. Therefore, radiation-induced c-Jun phosphorylation depends on the canonical MEK-ERK signaling pathway. Further definition of function for other components in this signaling pathway will depict a more complete picture of radiation-induced c-Jun activation.

ROS generation is one of the earliest cellular responses after ionizing radiation [46]. Radiation-caused ROS can be generated through oxidation of cellular water, leakage of the mitochondrial electron chain, or elevated activation of NADPH oxidases [2], [26]. The increased radicals and ROS, including superoxides, hydrogen peroxide, and hydroxyl radicals, can attack neighbor cells and alter gene transcription, consequently leading to cellular lesions and other responses such as DNA damage and inflammation. Therefore, ROS production has been thought to be a critical factor associated with radiation-caused acute and chronic brain injury [2]. However, the exact molecular mechanisms by which radiation-induced oxidative stress mediates brain injury are so far insufficiently understood. In this regard, our study provided new insights into radiation-induced microglial activation. Our experimental data suggest that elevated ROS level following irradiation likely triggers the activation of ERK signaling in microglial cells, which can eventually control inflammatory gene expression through the modulation of c-Jun transcriptional activity.

We found that radiation did not increase expression and activation of RelA/p65, an important component of NF-kB signaling pathway, in the irradiated BV2 cells, and the MEK inhibitor had no effect on RelA/p65 (Figure S5). However, we demonstrated that the activated ERK/c-Jun signaling pathway is essential to the radiation-mediated induction of several proinflammatory genes in microglial cells. Many studies have shown that the MAPK pathway and c-Jun phosphorylation of Ser63 and Ser73 can be stimulated by LPS and other proinflammatory factors, such as TNFα and interleukins [19], [47], [48], [49]. We speculate that the accumulation of these proinflammatory factors may modulate radiation-induced c-Jun phosphorylation to further enhance c-Jun activation. Combined with previous studies, our findings suggest that c-Jun acts as a signaling amplifier through a positive feedback mechanism to augment inflammatory gene expression and consequently promotes radiation-caused neuroinflammation. Taken together, our study demonstrated the involvement of the MEK/ERK/c-Jun signaling pathway in radiation-induced microglial inflammation responses and suggested that clinical interventions using this information may help minimize side effects caused by brain radiation therapy.

## Materials and Methods

### Reagents and antibodies

ERK inhibitor [3-(2-Aminoethyl)-5-(4-ethoxyphenyl) methylene)-2,4-thiazolidinedione, HCl, Calbiochem Cat #328006], U0126 and SP600125 were purchased from Calbiochem (EMD Biosciences, La Jolla, CA). The antioxidant N-acetyl-L-cysteine (NAC) was purchased from Sigma-Aldrich Corporation (St. Louis, MO). The antibodies for JNK (#9258), phosphorylated JNK (Thr/183/Thr185, #9255), cyclin D1 (#2926) and phosphorylated c-Jun (Ser63, #2361) were purchased from Cell Signaling Technology (Beverly, MA). Antibodies for c-Jun (sc-45), phosphorylated c-Jun (Ser73, sc-7981), ERK1 (sc-94), phosphorylated ERK1/2 (Thr202/Thr204, sc-81492), phosphorylated MEK (sc-7995R), COX-2 (sc-1745) and HA (sc-7392) were purchased from Santa Cruz Biotechnology (Santa Cruz, CA). MEK antibody (RB-1662) was purchased from Thermo Scientific (Waltham, MA).

### Cell cultures, transfection and treatments

Murine microglial BV2 cells [50] were cultured in high glucose DMEM medium supplemented with 5% fetal bovine serum (FBS) at 37°C with 10% CO_2_. Cells were transiently transfected with varied amounts of plasmids using Fugene HD (Roche Applied Science) according to the manufacturer's instruction. Forty-eight hours post transfection, the cells were subjected to further analysis. BV2 cells were used under passage 30. In the kinase inhibitor assay, the inhibitors were supplemented to culture medium 1 h before exposure to irradiation. For radiation treatment, cells were starved with serum-free DMEM medium for 24 h prior to irradiation. Cells were irradiated with a ^137^Cs irradiator (J.L. Shepherd and Associates, San Fernando, CA) at a dose rate of 3.7 Gy/min. All irradiations were performed at room temperature; control cells were received sham irradiation. After irradiation, the culture dishes were returned to the incubator.

### DNA constructs

In this study, murine cDNAs of c-Jun, ERK1, ERK2, and MEK1 were cloned into appropriate expression vectors, and the shRNA constructs were designed to target murine mRNA sequences. The expression plasmids pCMV-SPORTS6/ERK1 and pCMV-SPORTS6/ERK2 were obtained from Open Biosystems (Thermo Scientific, Waltham, MA). The coding regions of c-Jun and MEK1 were amplified with RT-PCR using cDNA from BV2 cells and subcloned into different vectors. The kinase-dead MEK1 (MEK1^K97M^) [24] was created by PCR-based site-directed mutagenesis. pSL2/HA vector [28] was used to overexpress HA-tagged wild-type and the mutant MEK1 in mammalian cells. To bacterially express ERK1, ERK2 and c-Jun mutants, the corresponding coding fragments were inserted into vectors to form glutathione S-transferase (GST)-tagged or 6xHis-tagged fusion proteins. The c-Jun truncation mutants, including aa1-62, aa1-190, and aa191-334, were generated by PCR methods. To achieve efficient gene knockdown, lentivirus-based shRNAs were delivered into BV2 cells by infection. shRNA expression vectors were constructed on the lentiviral vector pLu containing mouse U6 promoter as detailed previously [28]. The target sequences used for shRNA construction are sh-ERK1-536 (gcgaccttaagatctgtgatt), sh-ERK1-974 (gcgcatcacagtagaggaagc), sh-ERK2-321 (gacggacctttacaagctctt), sh-ERK2-772 (gctagaaactatttgctttctc), sh-MEK1-212 (ggatgatgactttgagaagat), and sh-MEK1-495 (ggaagaattcctgagcaaatt). The scrambled control was described elsewhere [51]. The shRNA plasmids were constructed using a methodology described previously [52]. The primer sequences are detailed in the Table S1.

### GST pull-down assay and co-immunoprecipitation

GST pull-down assays were performed as described previously [51]. Co-immunoprecipitation was carried out to detect the endogenous interaction between phosphorylated ERK1/2 and c-Jun. 750 µg of BV2 cell lysates were incubated with c-Jun polyclonal antibody (sc-45; Santa Cruz) for 2 h at 4°C in a binding buffer [0.05 M Tris-HCl (pH 7.5), 0.15 M NaCl, 5 mM EDTA, 0.1% NP-40] containing PSFM and 1x protease inhibitor cocktail (Roche Applied Science), followed by incubation with protein G-agarose (Invitrogen, Carlsbad, CA) for 2 h. After extensive washing with the binding buffer, the samples were analyzed by Western blot using the p-ERK1/2 antibody (sc-81492) and ERK1 antibody (sc-94), respectively.

### Lentiviral infection

shRNA lentiviral production was performed by co-transfecting 293FT cells with lentiviral shRNA vectors together with the packaging plasmids: pVSV-G, pRSV-REV, and pMDLg/pRRE. The medium was filtrated with a 0.45 μ filter 48 h post-transfection and then ultracentrifuged (25,000 rpm for 1.5 h) to concentrate viral particles. To infect BV2 cells, the medium was added with concentrated lentivirus and polybrene (8 mg/ml) and incubated for 6 h before replacing with normal medium.

### Real-time PCR

The primers used for real-time PCR were: mTNFa (F: CACCACCATCAAGGACTC/R: AGGTCTGAAGGTAGGAAGG), mIL-1b (F: ATCCGTGTCTTCCTAAAGTATG/R: CCTGAGCGACCTGTCTTG), mCOX-2 (F: TGTCTTCCAGCCCATTGAAC/R: CAGCGTTTCTCGTAGTAAAGTG), mIL-6 (F: GCTGGAGTCACAGAAGGAG/R: GAGAACAACATAAGTCAGATACC), and GAPDH (F: GCCTTCCGTGTTCCTACC/R: CTTCACCACCTTCTTGATGTC). Total RNA was prepared by Trizol (Invitrogen, Carlsbad, CA) method, and further purified with the RNeasy Mini kit (Qiagen, Santa Clarita, CA) after DNase I treatment. Reverse transcription was performed with MMLV (Invitrogen, Carlsbad, CA) following the manufacturer's instructions. Real-time PCR was performed at ABI7000 using FastStart universal SYBR green master mix (Rox) (Roche Applied Science, Mannheim, Germany). The samples were analyzed in quadruplicate and repeated in three independent experiments. The comparative *C*_t_ method was used to calculate relative levels of gene expression based on normalization to GAPDH.

### Electromobility shift assay (EMSA)

Nuclear protein was extracted from treated and untreated BV2 cells following a procedure described previously [53]. Protein concentrations were determined by a BCA kit (Pierce, Rockford, IL). The AP-1 probe consisted of annealed oligonucleotides of the consensus AP-1-binding sequence (5′-CGCTTGATGAGTCAGCCGGAA-3′ and 3′-GCGAACTACTCAGTCGGCCTT-5′), which was labeled with γ-^32^P-ATP (Perkin Elmer Life and Analytical Sciences, Waltham, MA) using T4 polynucleotide kinase (Promega, Madison, WI). The labeled probe was cleaned with a Sephadex G-25 column (GE Healthcare) to remove the free γ-^32^P-ATP and radioactivity was quantified using the LS6500 liquid scintillation counter (Beckman Coulter, Fullerton, CA). The EMSA procedure was performed by using the Gel-Shift Core Assay Kit (Promega, Madison, WI) with slight modifications. Briefly, 10 μg of nuclear protein was incubated with labeled AP-1 probe (∼150 cpm/reaction) at 37°C for 1 h. The binding products were then resolved on a 5% native polyacrylamide gel (0.5× TBE). The gel was subsequently vacuum-dried (Thermo Scientific, Waltham, MA) for 2 h at 85°C before autoradiography.

### Statistical analysis

All experimental data were collected, unless otherwise indicated, from 3 independent experiments, and presented as mean ± SD. Statistical analysis was performed with SigmaPlot 10.0 (Systat Software, San Jose, CA) and Student's *t* test was used to evaluate significance of differences between two groups. A *P* value of <0.05 was considered statistically significant.

## Supporting Information

Figure S1 **Protein interaction between c-Jun and ERK1/2 in cells.** A lentivirual vector pSL2 was used to construct overexpression vectors for HA-ERK1, HA-ERK2, and Flag-c-Jun. BV2 cells were infected with the lentiviruses to express the indicated proteins. Forty-eight hours after infection, cell lysates were subjected to immunoprecipitation assay with the anti-Flag antibody (sc-807, Santa Cruz Biotech) and control antibody, and then the HA monoclonal antibody (sc-7392, Santa Cruz Biotech) was used to detect the precipitated HA-ERK1 or HA-ERK2 in a Western blot assay. To show binding specificity, the cell lysates expressing HA-ERK1 or HA-ERK2 alone were included in the co-immunoprecipitation assay. The expression of Flag-c-Jun, HA-ERK1, HA-ERK2, and GAPDH (loading control) in the co-immunoprecipitation lysates is shown in the lower panel, respectively.
(PDF)

Figure S2 **The relative levels of p-c-Jun and p-ERK1/2 in Western blots normalized against the levels of their total proteins.** Quantification was performed with the Quantity One 1-D Analysis software (Bio-Rad, Richmond, CA). (A) and (B) Quantification results of p-c-Jun and p-ERK1/2 in U0126 inhibitor assay shown in Figure 4C. (C) and (D) Quantification results of p-c-Jun and p-ERK1/2 in MEK knockdown experiment shown in Figure 4D. Data represent mean ± SD, *p<0.05; **p<0.01.
(PDF)

Figure S3 **Ser63 and 73 are critical for c-Jun activity in BV2 cells.** (A) Schematic diagram for 4XUAS reporter and Gal4-c-Jun fusion constructs. 4XUAS was amplified from 4XUAS-TK-Luc [29] and inserted into the pGLuc-Basic vector (New England Biolabs) containing the Gaussia luciferase reporter gene. The indicated partial c-Jun containing transcription activation (TA) domain was used to detect c-Jun transcriptional activity. Three mutants were created for c-Jun (TA), in which Ser63 and Ser73 were replaced with alanine separately or together. The resulting c-Jun variants were in frame fused with the Gal4 DNA binding domain (DBD) in the pcDNA3 vector. (B) BV2 cells were cotransfected with 75 ng Gal4-c-Jun (TA) and its mutants, together with 300 ng 4xUAS-Gluc reporter and 100 ng SEAP expression vector as an internal control. Cells were irradiated with 10 Gy 30 h after transfection and medium were collected 4 h after radiation treatment for measuring luciferase activity. The measurement method was detailed previously [28]. The relative reporter activities were normalized against SEAP activity. Experiments were performed twice in triplicate. Data represent mean ± SD, and differences were evaluated between the effects of wild-type and mutant c-Jun, *p<0.05.
(PDF)

Figure S4 **Model for radiation-induced c-Jun phosphorylation and its role in neuroinflammation.**
(PDF)

Figure S5 **Radiation does not induce NFkB activation in BV2 cells.** (A) RelA/p65 level in the irradiated BV2 cells. (B) Levels of nuclear form of RelA/p65 in irradiated BV2 cells with or without MEK inhibitor U0126 treatment. Histone H3 and Ponceau S staining represent loading controls for the nuclear extracts.
(PDF)

Table S1 **Primers for cloning.**
(DOC)
