# Supplementary figures and images for: Radiation-Induced c-Jun Activation Depends on MEK1-ERK1/2 Signaling Pathway in Microglial Cells
Source: PLoS One. 2012 May 14;7(5):e36739. doi: 10.1371/journal.pone.0036739 (PMC3351464; doi:10.1371/journal.pone.0036739)

Figure S1

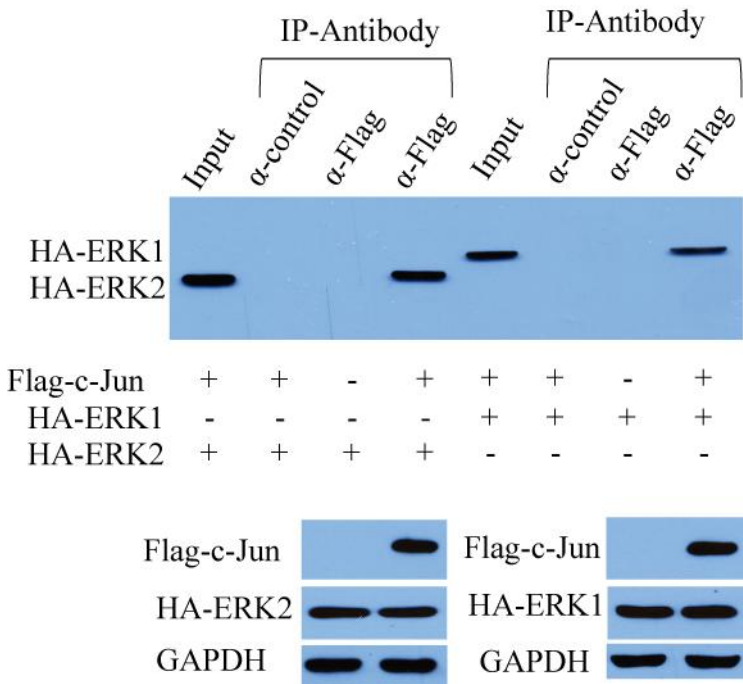

Supplement: Figure S1 — Protein interaction between c-Jun and ERK1/2 in cells. A lentivirual vector pSL2 was used to construct overexpression vectors for HA-ERK1, HA-ERK2, and Flag-c-Jun. BV2 cells were infected with the lentiviruses to express the indicated proteins. Forty-eight hours after infection, cell lysates were subjected to immunoprecipitation assay with the anti-Flag antibody (sc-807, Santa Cruz Biotech) and control antibody, and then the HA monoclonal antibody (sc-7392, Santa Cruz Biotech) was used to detect the precipitated HA-ERK1 or HA-ERK2 in a Western blot assay. To show binding specificity, the cell lysates expressing HA-ERK1 or HA-ERK2 alone were included in the co-immunoprecipitation assay. The expression of Flag-c-Jun, HA-ERK1, HA-ERK2, and GAPDH (loading control) in the co-immunoprecipitation lysates is shown in the lower panel, respectively. (PDF) [file pone.0036739.s001.pdf]

Figure S2

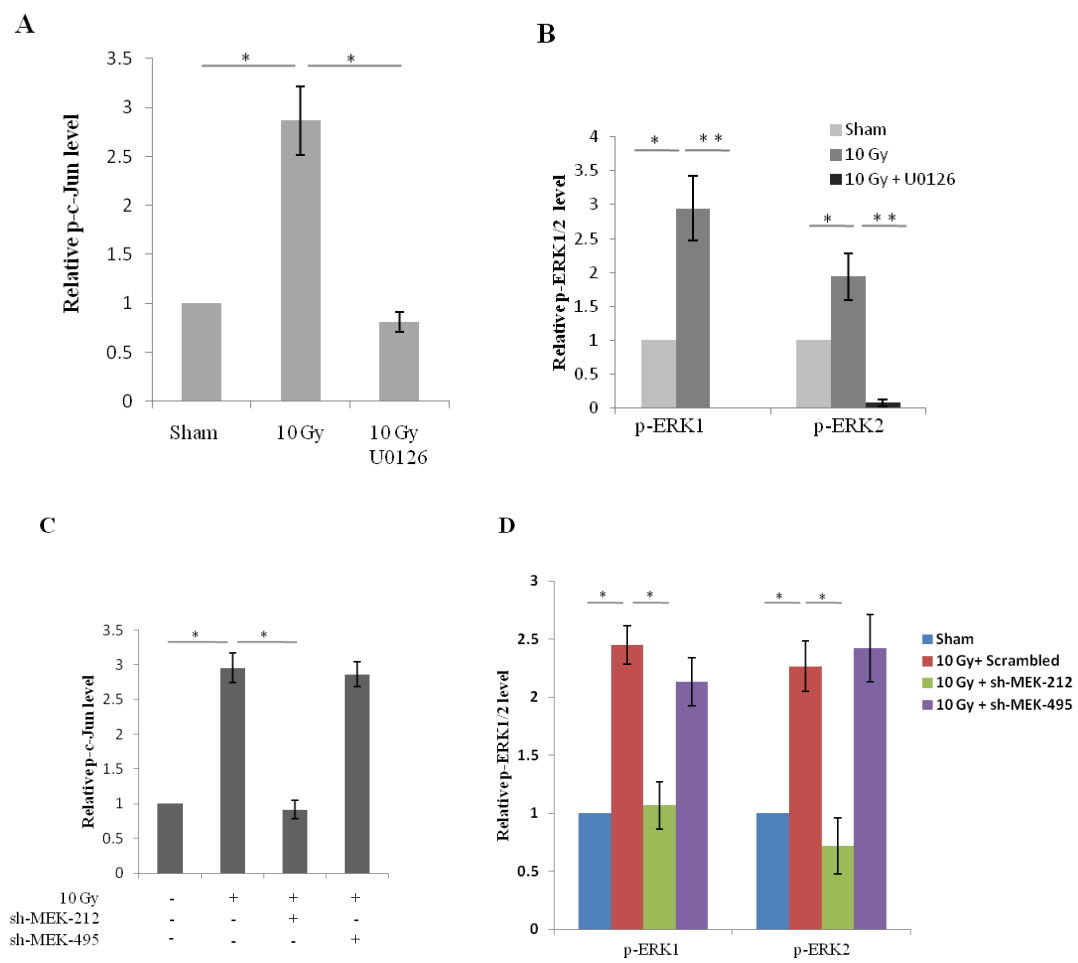

Supplement: Figure S2 — The relative levels of p-c-Jun and p-ERK1/2 in Western blots normalized against the levels of their total proteins. Quantification was performed with the Quantity One 1-D Analysis software (Bio-Rad, Richmond, CA). (A) and (B) Quantification results of p-c-Jun and p-ERK1/2 in U0126 inhibitor assay shown in Figure 4C. (C) and (D) Quantification results of p-c-Jun and p-ERK1/2 in MEK knockdown experiment shown in Figure 4D. Data represent mean ± SD, *p<0.05; **p<0.01. (PDF) [file pone.0036739.s002.pdf]

**Figure S3****A**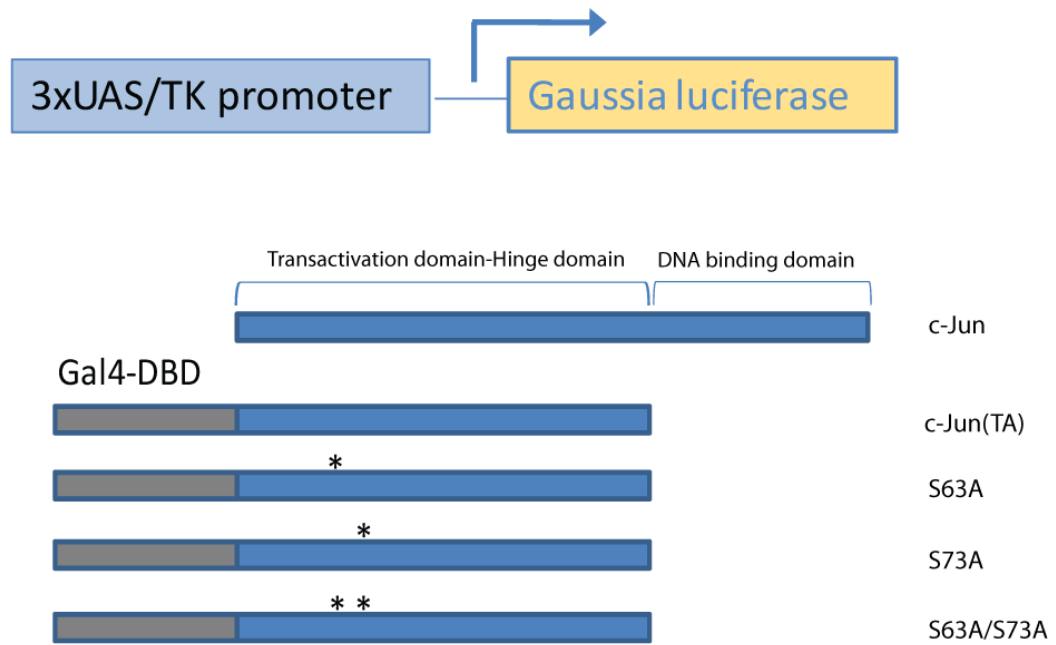**B**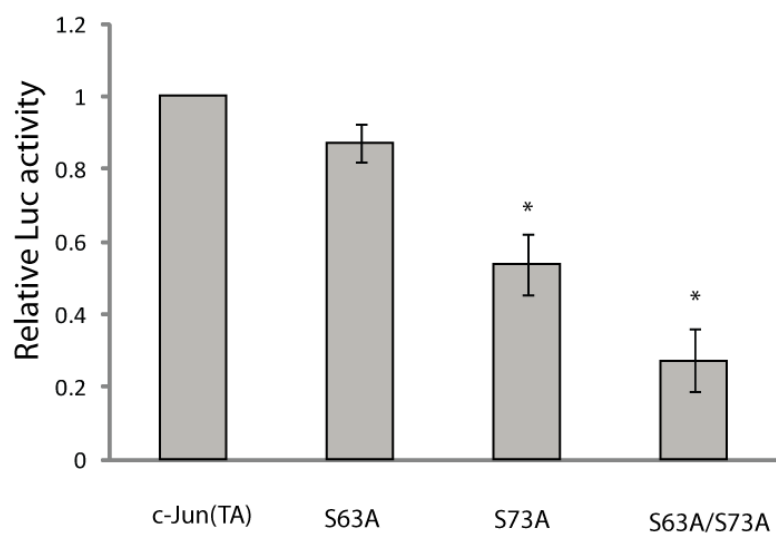

Supplement: Figure S3 — Ser63 and 73 are critical for c-Jun activity in BV2 cells. (A) Schematic diagram for 4XUAS reporter and Gal4-c-Jun fusion constructs. 4XUAS was amplified from 4XUAS-TK-Luc [29] and inserted into the pGLuc-Basic vector (New England Biolabs) containing the Gaussia luciferase reporter gene. The indicated partial c-Jun containing transcription activation (TA) domain was used to detect c-Jun transcriptional activity. Three mutants were created for c-Jun (TA), in which Ser63 and Ser73 were replaced with alanine separately or together. The resulting c-Jun variants were in frame fused with the Gal4 DNA binding domain (DBD) in the pcDNA3 vector. (B) BV2 cells were cotransfected with 75 ng Gal4-c-Jun (TA) and its mutants, together with 300 ng 4xUAS-Gluc reporter and 100 ng SEAP expression vector as an internal control. Cells were irradiated with 10 Gy 30 h after transfection and medium were collected 4 h after radiation treatment for measuring luciferase activity. The measurement method was detailed previously [28]. The relative reporter activities were normalized against SEAP activity. Experiments were performed twice in triplicate. Data represent mean ± SD, and differences were evaluated between the effects of wild-type and mutant c-Jun, *p<0.05. (PDF) [file pone.0036739.s003.pdf]

Figure S4

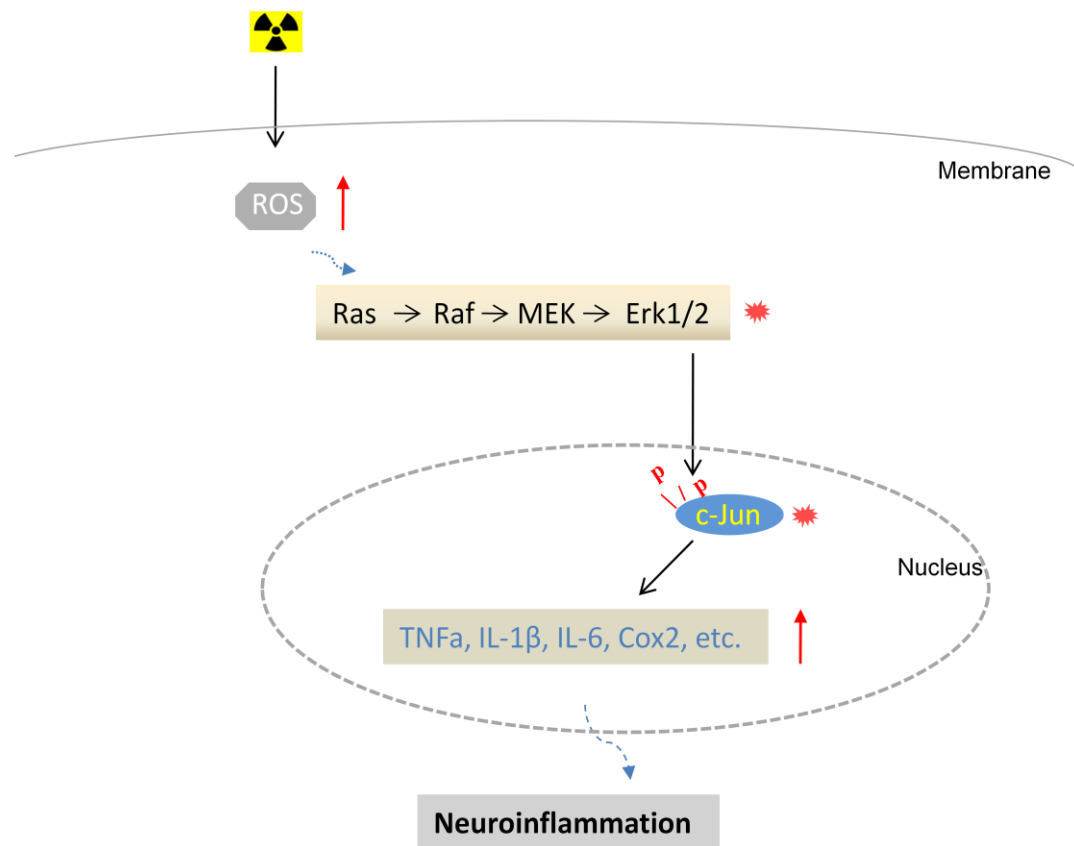

Supplement: Figure S4 — Model for radiation-induced c-Jun phosphorylation and its role in neuroinflammation. (PDF) [file pone.0036739.s004.pdf]

**Figure S5**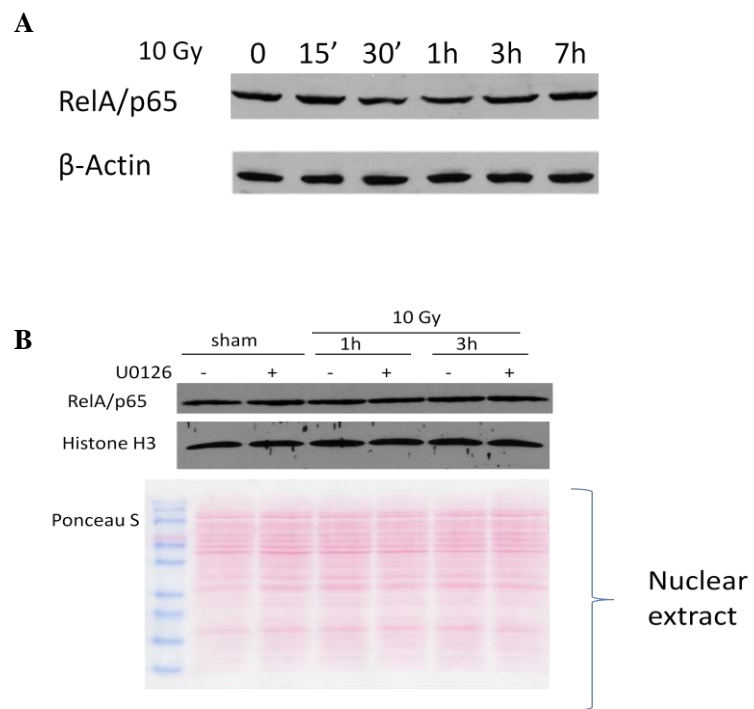

Supplement: Figure S5 — Radiation does not induce NFkB activation in BV2 cells. (A) RelA/p65 level in the irradiated BV2 cells. (B) Levels of nuclear form of RelA/p65 in irradiated BV2 cells with or without MEK inhibitor U0126 treatment. Histone H3 and Ponceau S staining represent loading controls for the nuclear extracts. (PDF) [file pone.0036739.s005.pdf]
