# Supplementary material for: Radiation-Induced c-Jun Activation Depends on MEK1-ERK1/2 Signaling Pathway in Microglial Cells
Source: PLoS One. 2012 May 14;7(5):e36739. doi: 10.1371/journal.pone.0036739 (PMC3351464; doi:10.1371/journal.pone.0036739)
Supplement: Table S1 — Primers for cloning. (DOC) [file pone.0036739.s006.doc]

| **Name** | **Sequence** | **Usage** |
| --- | --- | --- |
| Mus-cJun-Bm-4U | tcgggatccactgcaaagatggaaacgacc | 6xHis or GST-c-Jun |
| Mus-cJun-XbaI-1005L | tagtctagatcaaaacgtttgcaactgctg |
| Mus-Erk1-Bm-4U | tcgggatccgcggcggcggcggcggctccg | 6xHis or GST-ERK1 |
| Mus-Erk1-XbaI-1143L | tagtctagattaggggccctctggcgcc |
| Mus-Erk2-Bm-4U | tcgggatccgcggcggcggcggcggcgggc | 6xHis or GST-ERK2 |
| Mus-Erk2-XbaI-1077L | tagtctagattaagatctgtatcctggctgg |
| Mus-MEK1-Bm-4U | tcgggatcccccaagaagaagccgacgccc | pSL2-HA-MEK1 |
| Mus-MEK1-XbaI-1182L | tagtctagatcagatgctggcagcgtgg |
| Mus-MEK1^K97M^-L | cataactagtccagatggcttgtgggaga | HA-MEK1^K97M^ |
| Mus-MEK1^K97M^-U | ctggactagttatggctagaatgctgatc |
| Mus-cJUN-XbaI-570-L | tagtctagattaataggagggcgccccaccgc | c-Jun (aa1-190) |
| Mus-cJUN-XbaI-186-L | tagtctagacgtgagaaggtccgagttct | c-Jun (aa1-62) |
| Mus-cJun-Bm-591-U | tcgggatccggcgcggccgggctggccttt | c-Jun(aa191-334) |
| Mus-Erk1-536-H3-L | agcaagcttgaatatcacagatcttaaggtcgcaaacaaggcttttctccaagggata | sh-ERK1-536 |
| Mus-Erk1-536-2a | agcttaatcacagatcttaaggtcgctttttg |
| Mus-Erk1-536-2b | aattcaaaaagcgaccttaagatctgtgatta |
| Mus-Erk1-974-H3-L | agcaagcttgaatcttcctctactgtgatgcgcaaacaaggcttttctccaaggga | sh-ERK1-974 |
| Mus-Erk1-974-2a | agcttagcttcctctactgtgatgcgctttttg |
| Mus-Erk1-974-2b | aattcaaaaagcgcatcacagtagaggaagcta |
| Mus-MEK1-212-H3-L | cagcaagcttgaattcttctcaaagtcatcatccaaacaaggcttttctccaagggata | sh-MEK1-212 |
| Mus-MEk1-212-2a | agcttatcttctcaaagtcatcatcctttttg |
| Mus-MEk1-212-2b | aattcaaaaaggatgatgactttgagaagata |
| Mus-MEK1-495-H3-L | cagcaagcttgaatatttgctcaggaattcttccaaacaaggcttttctccaagggata | sh-MEK1-495 |
| Mus-MEk1-495-2a | agcttaatttgctcaggaattcttcctttttg |
| Mus-MEk1-495-2b | aattcaaaaaggaagaattcctgagcaaatta |

Table S1 Primers for cloning

| **Name** | **Sequence** | **Usage (Con’t)** |
| --- | --- | --- |
| Mus-Erk2-321-H3-L | cagcaagcttgaatagagcttgtaaaggtccgtcaaacaaggcttttctccaagggata | sh-ERK2-321 |
| Mus-Erk2-321-2a | agcttaagagcttgtaaaggtccgtctttttg |
| Mus-Erk2-321-2b | aattcaaaaagacggacctttacaagctctta |
| Mus-Erk2-772-H3-L | cagcaagcttgaacagaaagcaaatagtttctagcaaacaaggcttttctccaagggata | sh-ERK2-772 |
| Mus-Erk2-772-2a | agcttgagaaagcaaatagtttctagctttttg |
| Mus-Erk2-772-2b | aattcaaaaagctagaaactatttgctttctca |
